# Expression of NMDA receptor-dependent LTP in the hippocampus: bridging the divide

**DOI:** 10.1186/1756-6606-6-5

**Published:** 2013-01-22

**Authors:** Tim VP Bliss, Graham L Collingridge

**Affiliations:** 1 Division of Neurophysiology, National Institute for Medical Research, Mill Hill, London NW7 1AA, UK; 2 Centre for Synaptic Plasticity, School of Physiology and Pharmacology, University of Bristol, Bristol BS8 1TD, UK; 3 Department of Brain & Cognitive Sciences, College of Natural Sciences, Seoul National University, Rm 330, Bldg. 501 Shilim, Gwanak, Seoul, 151-746, Korea

## Abstract

A consensus has famously yet to emerge on the locus and mechanisms underlying the expression of the canonical NMDA receptor-dependent form of LTP. An objective assessment of the evidence leads us to conclude that both presynaptic and postsynaptic expression mechanisms contribute to this type of synaptic plasticity.

## Introduction

The view that the expression of N-methyl-D-aspartate (NMDA) receptor-dependent long-term potentiation (LTP) is achieved largely, if not exclusively, by purely postsynaptic mechanisms, involving the trafficking and insertion of AMPA receptors, has been promoted in a series of reviews and papers [1-3]. It remains our contention, as advanced previously [4,5], that a balanced assessment of the available evidence leads to the conclusion that in some experimental situations presynaptic mechanisms play an important, even a dominant role. Conversely, in other situations, it is clear that the expression of LTP is largely, if not exclusively, postsynaptic.

**Figure 1:**
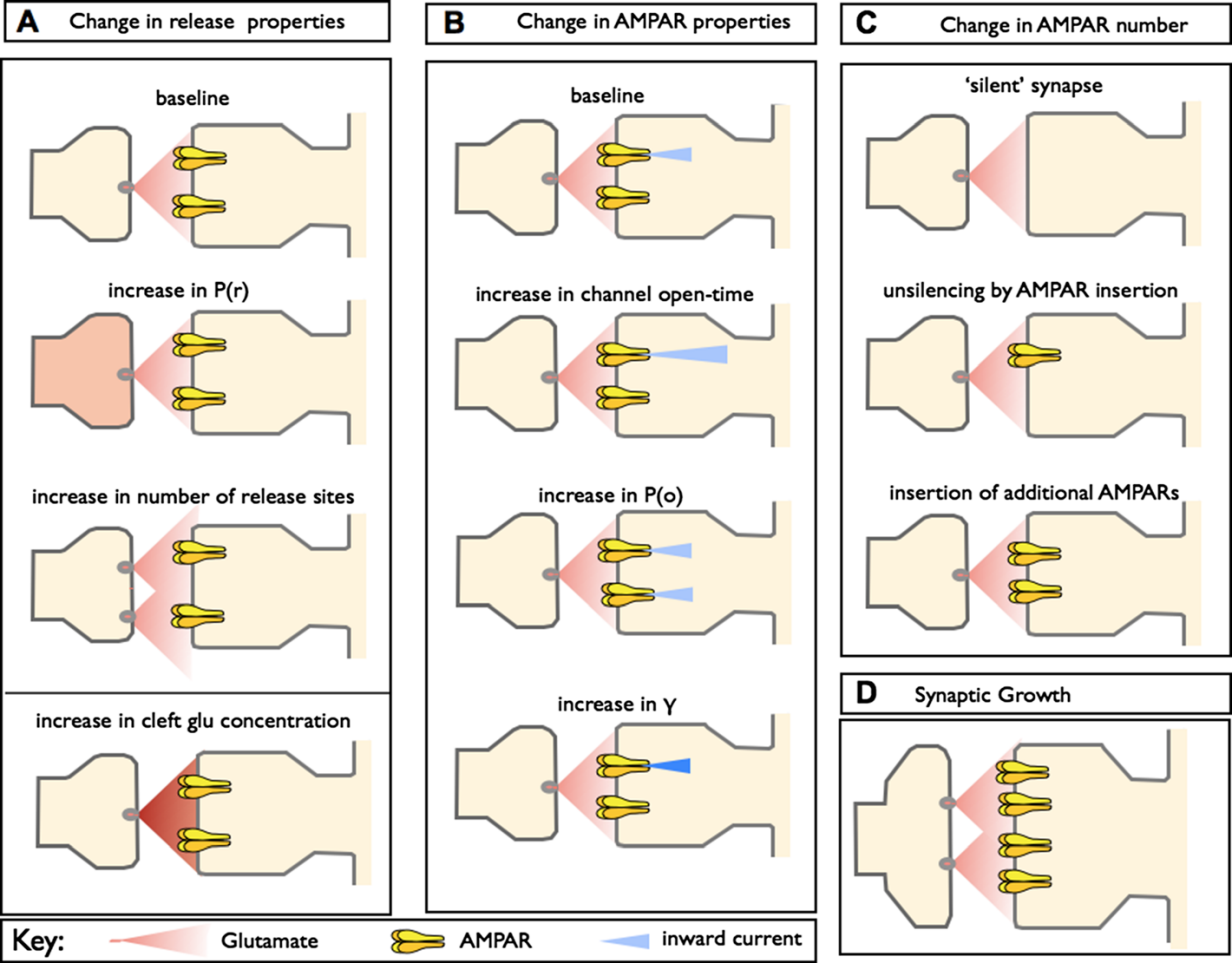
**Potential expression mechanisms for LTP. A. **LTP could involve presynaptic mechanims due to an increase in p(r), number of release sites (n) or cleft glutamate (glu) concentration. **B. **Postsynaptic mechanisms could involve a modification of AMPA receptor properties, such as an increase in their mean open-time, probability of opening on binding glutamate p(o), or an increase in their single channel conductance (γ). **C. **Postsynaptic mechanisms could also involve an increase in the number of AMPA receptors at synapses. **D. **Synaptic growth is likely to involve both pre and postsynaptic changes.

The term LTP embraces a family of plasticity-related phenomena, including tetanus-induced LTP, pairing-induced LTP, spike-timing dependent LTP and chemically-induced LTP, each with potentially distinct expression mechanisms. To add to the complexity, LTP can be induced in a wide variety of neural pathways over a range of developmental ages and in a number of different *in vitro* and *in vivo* preparations. In most cases, LTP has been followed for no more than an hour or so, and in no case is there sufficient information to give a complete account of the expression mechanism at all times following induction. Most of the experiments that have addressed the question of the locus of expression have been performed on hippocampal slices, usually by studying AMPA receptor-mediated synaptic transmission at the Schaffer collateral - commissural pathway that connects the CA3 and CA1 pyramidal cell fields. In this review we examine the literature relevant to the locus of expression of NMDA receptor-dependent LTP (referred to hereafter simply as LTP). We consider the range of possible expression mechanisms shown schematically in Figure 1. Note than in many cases the experimental techniques adopted can address only one side of the debate - for instance, measurement of glutamate release cannot throw light on whether or not there are postsynaptic changes, and conversely analysis of glutamate receptor trafficking can have nothing to say about presynaptic changes. Other techniques are in principle capable of providing evidence for pre or postsynaptic changes (for example, quantal analysis).

## Presynaptic mechanisms

We begin with a summary of results which have yielded evidence for presynaptic changes.

### Glutamate release studies

The first direct evidence for a presynaptic locus of expression of LTP came from experiments that correlated LTP with changes in the concentration of extracellular glutamate. This approach used push-pull perfusion [6,7] in the dentate gyrus *in vivo* to reveal an increase in extracellular glutamate concentration that was NMDA receptor-dependent and persisted for at least two hours after induction of LTP. (A failure to reproduce this finding suffers from a lack of a positive control [8]). The possibility that presynaptic mechanisms may contribute to longer-lasting forms of synaptic plasticity is suggested by the finding that an increase in glutamate release was also detected in hippocampal slices prepared from animals in which LTP had been induced several days previously [9]. These early studies measured total levels of glutamate in the perfusate. However, a later study, using glutamate-sensitive electrodes [10], measured the increase in glutamate concentration in response to an increase in the frequency of synaptic stimulation and this activity-dependent component of the signal was potentiated following the induction of LTP. These studies, whilst lacking the spatial or temporal resolution to identify the precise source of the increase in release, clearly demonstrated the potential for presynaptic expression mechanisms in LTP.

### Paired-pulse facilitation

**Figure 2:**
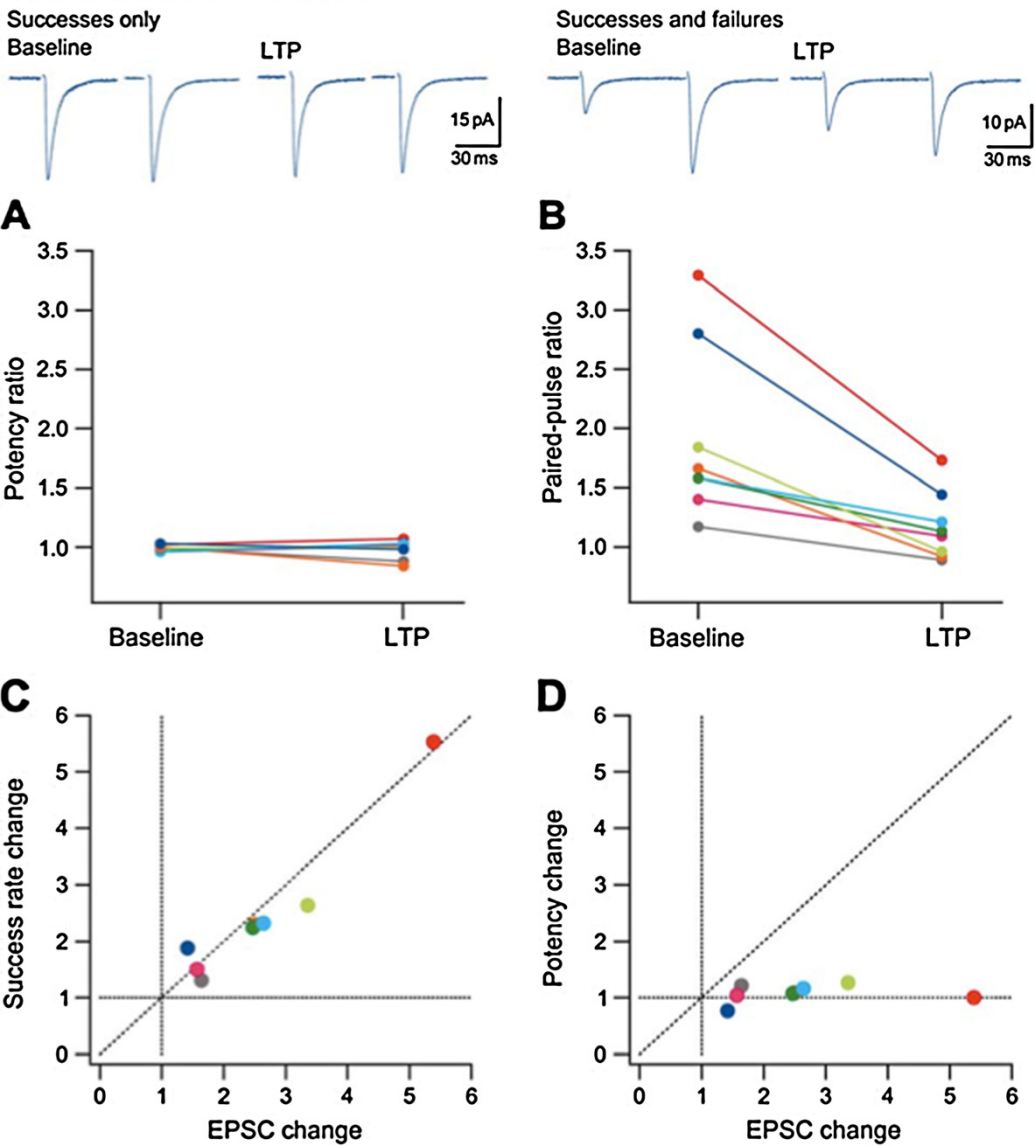
**Evidence for presynaptic changes in LTP. **When a single fiber is stimulated, a change in success rate without change in potency establishes that the alteration is presynaptically mediated. (**A**) A plot of paired-pulse potency (mean amplitude excluding failures) ratio during baseline and following the induction of LTP for 8 neurons. (**B**) A plot of paired-pulse ratio (mean amplitude including failures) during baseline and following the induction of LTP for the same neurons. Note that despite large PPF during baseline and, for some neurons, following the induction of LTP the paired-pulse potency ratio is approximately 1 both during baseline and following the induction of LTP. (**C**) A plot of success rate change vs amplitude change for LTP of these neurons. (**D**) The corresponding plot for potency. Note that for all neurons the increase in EPSC amplitude can be explained by the success rate change and that potency is unaltered. From [25].

A technique for distinguishing pre from postsynaptic changes based on the phenomenon of paired-pulse facilitation (PPF) was introduced by McNaughton [11] and has been widely adopted as a rough and ready test. In PPF (at multiple synapses), the second of a pair of stimuli separated by a few tens of milliseconds produces a larger synaptic response than the first, owing to the transient elevation of Ca^2+^ in terminal boutons produced by the second stimulus summing with the residual Ca^2+^ produced by the first stimulus, and increasing the probability of transmitter release, p(r). Since p(r) cannot exceed 1, the PPF ratio tends to decrease as p(r) increases. This is what happens in the presynaptic phenomenon of post-tetanic potentiation, a large and transient increase in the synaptic response that immediately follows tetanic stimulation of most monosynaptic excitatory pathways in the hippocampus. In experiments on the lateral perforant path in the anaesthetised rat, McNaughton [11] observed the expected decrease in PPF immediately after tetanic stimulation, but as the potentiation declined over the subsequent few minutes, so PPF recovered to reach its pre-tetanus value during the sustained enhancement characteristic of LTP. Similar experiments have been performed many times at a variety of different hippocampal pathways with inconsistent results, some authors reporting decreases in PPF in LTP and others finding no change (see [5]). This variability is not simply a reflection of differences in experimental preparations; in at least one study, LTP was associated with pronounced PPF changes in some neurons and with no alterations in PPF in other neurons recorded under identical conditions [12]. Note, moreover, that postsynaptically mediated changes in PPF are also theoretically possible (see [5], Box 1 for an example). However, in cases where LTP is associated with a reduction in PPF, an increase in p(r) is the most straightforward explanation. An example of a large decrease in PPF accompanying LTP in a putative single-fibre experiment is shown in Figure 2. This result is difficult to explain other than by an increase in p(r) and demonstrates that in some circumstances analysis of PPF can lead to the conclusion that LTP has a presynaptic component.

### Vesicular fusion

Several groups have looked for changes in the frequency of vesicular fusion following the induction of LTP. Malgaroli et al. [13] used antibodies to the vesicular protein synaptotagmin to measure the rate of vesicle fusion in cultured primary neurons, and found that LTP was accompanied by a greater frequency of fusion events. Real-time methods of measuring the probability of release across a population of presynaptic terminals became available with the introduction of the fluorescent dye FM1-43 which readily penetrates the synaptic cleft and is taken up into vesicles during membrane fusion [14]. During a subsequent round of stimulation (‘destaining’), the rate at which fluorescence is lost from the presynaptic terminal reflects the frequency of vesicle fusion, and hence provides an estimate of p(r). Experiments based on this method have revealed an increase in the rate of destaining following the induction of LTP under certain conditions in primary hippocampal cultures [15], in slices from area CA1 [16,17] and in the low p(r) synapses made by the perforant path projection to distal apical dendrites of CA1 pyramidal cells [18]. Finally, in CA1 slices from transgenic mice expressing synaptopHluorin, the pH-sensitive GFP construct of the vesicular protein VAMP2, a delayed but sustained increase in synaptic vesicle recycling was observed following the induction of LTP [19]. Collectively this group of results constitute strong evidence that presynaptic changes in LTP involve sustained modifications of the release machinery.

### Quantal Analysis

Quantal analysis of LTP was attempted in many labs during the 1980s and 1990s, but gradually fell out of favour because of the difficulties of interpretation. The technique was first used by Fatt and Katz in their analysis of quantal transmission at the neuromuscular junction, where there is typically a single synapse consisting of many release sites and their corresponding postsynaptic specializations [20]. A hippocampal principal neuron, in contrast, contains thousands of synapses, each typically with a single release site. Nevertheless, some useful and informative measures carry over from one model to the other. As discussed above, the probability of release, p(r), at a hippocampal synapse refers to the probability that there will be a release event at the single release site following invasion of the bouton by an action potential. Quantal size is defined as the amplitude of the EPSP or EPSC recorded, usually at the cell body, following a single release event; this is sometimes called the potency of the synapse [21]. The variability of the synaptic response across a population of synapses can be used to generate a parameter 1/CV^2^ where CV is the coefficient of variation of the evoked compound EPSP or EPSC (CV=standard deviation/mean); in classical statistical models of quantal analysis, CV depends only on presynaptic factors (p(r) and n, the number of active release sites).

### Silent Synapses

An analysis of alterations in CV following the induction of LTP led to the proposal that there are silent synapses in the hippocampus [22]. Silent synapses are defined as those that do not elicit a synaptic response following the invasion of the presynaptic bouton by an action potential. A synapse can be presynaptically silent if it lacks active release sites or if p(r) is zero, or postsynaptically silent if it lacks AMPA receptors (see below). Schemes for how the postsynaptic activation of NMDA receptors might unsilence a presynaptically silent synapse have been suggested [23]. Evidence for another presynaptic mechanism for the unsilencing of silent synapses has been presented, based on a modification of the vesicular fusion pore [24]. Under baseline conditions, the slow release of glutamate from an incompletely fused vesicle may result in glutamate desensitizing rather than activating AMPA receptors. LTP-inducing stimulation leads to an increase in the probability of a full fusion event and hence to an increase in the number of EPSCs. In the same study, LTP was shown to be associated with an increase in cleft glutamate concentration, as probed with a weak competitive NMDA receptor antagonist.

### Increases in p(r)

A reduction in the failure rate following the induction of LTP has often been observed when minimal stimulation is employed to activate one or a small number of fibres. By classical quantal analysis this would be interpreted as an increase in p(r), but a reduction in the failure rate could also result from an increase in the number of active synapses by unsilencing. Studies using minimal stimulation in hippocampal slices have, however, produced some convincing examples of single fibre activation – as, for example, when PPF is associated with an increase in the amplitude of the second response averaged over several trials, without change in potency [12,25]. In such cases it is difficult to argue with the conclusion that the increase in mean amplitude of the evoked response to the second stimulus reflects an increase in p(r) at a single activated synapse. An increase in the number of successes without change in potency following the induction of LTP at putative single synapses has been reported in CA1 pyramidal cells in slices from 15-24d post-natal rats [21] and in a subset of CA1 cells recorded 6d postnatally [12,25] (Figure 2). These observations provide compelling evidence that under certain conditions presynaptic mechanisms mediate the expression of an early phase of LTP.

Insights into possible mechanisms underlying early developmental LTP have been obtained [26]. This form of LTP is associated with large changes in PPF, caused by the rapid loss of function of a presynaptic kainate receptor [26]. Before induction, a high affinity kainate receptor senses the ambient levels of glutamate and acts to keep p(r) low. Following induction of LTP, the affinity of these kainate receptors is greatly reduced with the result that the presynaptic terminal can no longer sense the ambient levels of glutamate and p(r) becomes much higher. The rapid switch may involve the activity-dependent loss of a high affinity kainate receptor subunit.

### Optical quantal analysis of LTP

Quantal analysis took on a new lease of life with the advent of imaging of synaptically induced calcium transients at single synaptic spines [27,28]. This approach has provided the most direct evidence so far for presynaptic changes in LTP [29-31]. The first experiments were performed on hippocampal organotypic cultures, and made use of the fluorescent Ca^2+^ indicator Oregon Green 488 to detect invasion of action potentials in presynaptic boutons, or stimulus-evoked excitatory post-synaptic calcium transients (EPSCaTs) in single dendritic spines. EPSCaTs are triggered by Ca^2+^ entry through NMDA receptor channels with the subsequent release of Ca^2+^ from intracellular stores [32]. The authors argued that EPSCaTs accurately report the success or failure of transmitter release at the presynaptic terminal following invasion by an action potential [32], and hence can be used to estimate p(r) at imaged spines. This technique was exploited in a subsequent paper to show that in most spines LTP was associated with an increase in p(r), measured 30 min after induction, and that this increase could be blocked by procedures that inhibited the induction of LTP [29]. This result constitutes strong evidence for a presynaptic component of LTP for the first 30–60 min following the induction of LTP in Schaffer collateral - CA1 synapses in organotypic hippocampal cultures.

**Figure 3:**
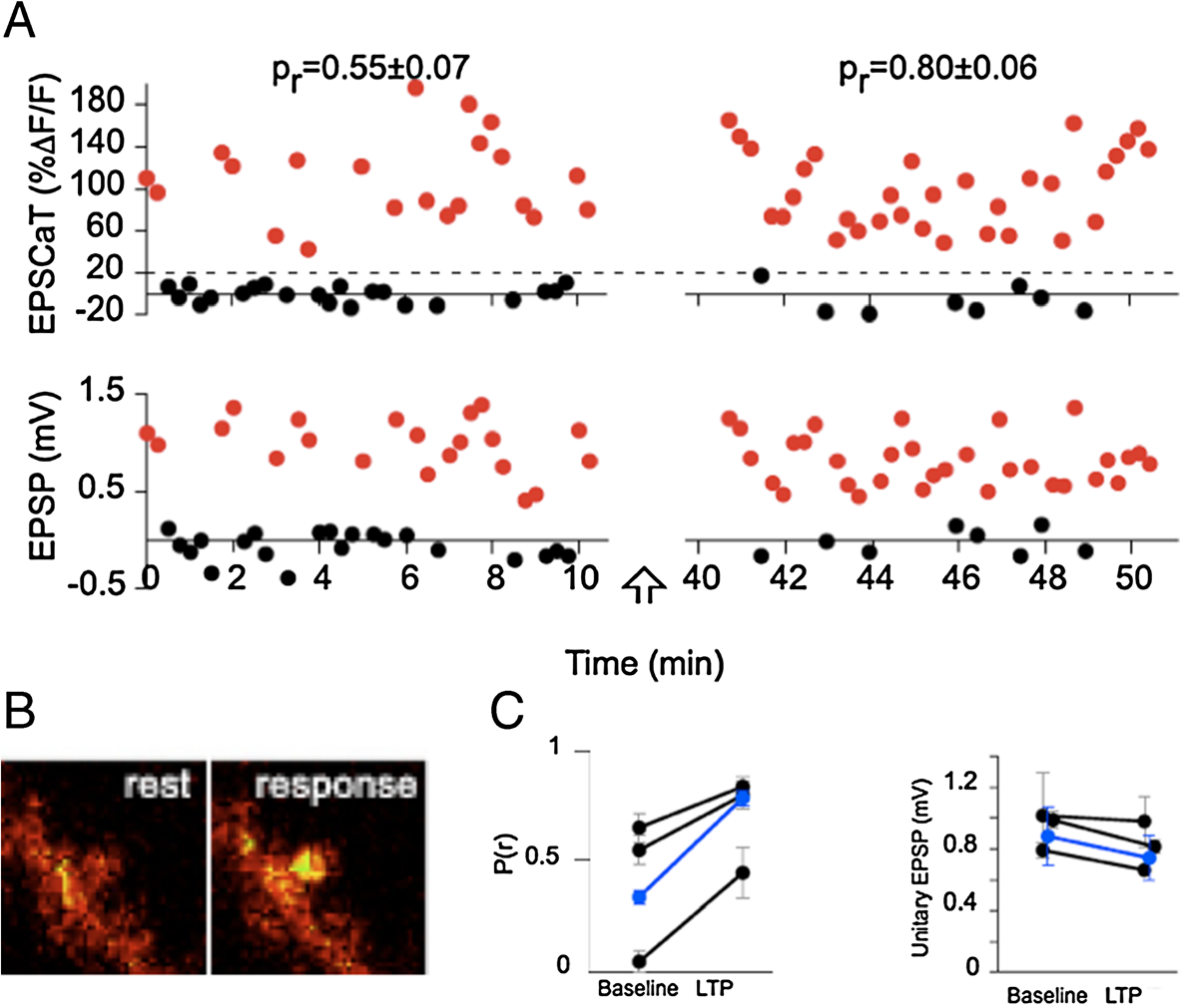
**Evidence for presynaptic changes in LTP. A. **Recordings of excitatory postsynaptic Ca^2+ ^transients (EPSCaTs, upper panels) in an activated spine, and simultaneously recorded somatic EPSPs (lower panels) from a neuron in which the imaged spine was the only one contributing to the synaptic response. LTP was associated with a large increase in p(r) with no change in quantal size. Red dots represent successes and black dots failures. **B. **Images of the spine at rest and during a response. **C. **Increase in p(r) without increase in potency for three neurons that fulfilled the criteria for a single activated synapse. From [30].

In a subsequent two photon study in acute hippocampal slices [30], Fine and his colleagues countered two concerns that the earlier confocal studies had raised: first, could the increase in probability of observing an EPSCaT following the induction of LTP reflect an increase in the coupling between the depolarization induced at the spine head when transmitter is bound and the release of calcium from internal stores? Second, do the results obtained from immature organotypic cultures carry over to the more mature acute slice? Enoki et al. [30] estimated the amplitude of the EPSP at the soma both by an indirect subtraction technique and directly in three experiments where they found an exact correspondence between the occurrence of the EPSCaT and the occurrence of the EPSP, establishing that the imaged spine was the only one on the recorded neuron that was responding to the stimulus (Figure 3). In these three experiments it was clear that the coupling between depolarization at the spine and the Ca^2+^ transient was completely secure both before and after the induction of LTP, and that p(EPSCaT) was an authentic reporter of p(r). In all three cases the induction of LTP resulted in an increase in p(r) without an increase in the potency of the synapse. A similar absence of change in potency was seen in the more usual case, where multiple synapses were activated and the potency of the imaged synapse was estimated by a subtraction technique. The direct monitoring of LTP at a single activated and imaged synapse establishes beyond reasonable doubt that there are synapses that can sustain early LTP by purely presynaptic mechanisms. A question that remains is how typical are such synapses? The process of scanning for responding spines inevitably biases the search towards high p(r) synapses that have the machinery to enable Ca^2+^-induced Ca^2+^ release. In some cells with multiple spines responding to the stimulus, Enoki et al. [30] carried out an ‘exhaustive scan’ of the dendritic arbor for all active spines to establish a lower limit for the number of such spines contributing to the EPSP, and estimated that the EPSCaT-producing spines contributed more than 40% of the EPSP recorded at the cell body. The estimation of average potency at these synapses was 0.63 mV, measured at the soma, significantly larger than that reported for CA1 cells by Sayer et al. [33], also using sharp electrodes (in the latter study the mean unitary EPSP recorded at the soma was 0.13 mV). But even if EPSCaT-producing spines are outnumbered by a greater number of low p(r) and/or low potency synapses, they are, nevertheless, major contributors to driving the activity of the cell.

In an accompanying commentary Lisman [34] accepted the compelling nature of these results and suggested, as had Enoki et al. [30], that they could be reconciled with postsynaptic accounts of plasticity if AMPA receptors are inserted into the periphery of the active zone, where they are likely to make little or no contribution to the EPSC. Lisman also considered the possibility that LTP is accompanied by a structural change leading to an increase in the number of release sites in the potentiated bouton; this would result in an increase in p(r) which would not necessarily be reflected by an increase in mean potency, even if there were insertion of extra AMPA receptors into the membrane, since the expected doubling of postsynaptic responses when release occurred from both old and new release sites would be attenuated by non-linear summation at the heavily depolarised spine. We are not convinced by this argument. Palmer and Stuart [35] estimate that the mean depolarisation at the spine following release of a single quantum of transmitter is 20 mV, which is still far from the reversal potential for AMPA receptor-mediated currents; moreover, the variance of single synaptic responses is over 100% ([30], Figure 3), also suggesting that the action of a single quantum is unlikely to take the spine to anywhere near its reversal potential.

Finally, a paper from the Emptage laboratory has introduced an independent method for measuring the locus of LTP in organotypic hippocampal cultures [31]. When the amplitude of the evoked Ca^2+^ transient was measured in Schaffer collateral terminals invaded by a series of action potentials, an unexpected bimodal distribution was found. McGuinness et al. established that the larger peak was the consequence of released transmitter binding to presynaptic NMDA receptors, resulting in an increase in the presynaptic Ca^2+^ signal. The enlarged Ca^2+^ response is thus a marker for transmitter release, and its frequency of occurrence can be used to estimate p(r). Exploiting this technique, McGuinness et al. confirmed that LTP was associated with an increase in p(r).

Imaging experiments tackle the issue of pre- vs post-synaptic expression of LTP in a notably direct manner. A single visualised spine is activated by the LTP-inducing stimulus, LTP is monitored at the soma, and in the case of the three spines imaged by Enoki et al. there is no ambiguity about the measurements of either p(r) or potency. This work, in our view, demonstrates beyond reasonable doubt that some CA1 synapses can generate a form of early LTP *in vitro* that is expressed wholly by presynaptic mechanisms.

## Postsynaptic mechanisms

There is widespread agreement that LTP is triggered by postsynaptically located NMDA receptors [4]. Numerous studies have shown that the manipulation of signalling molecules such as CaMKII in the postsynaptic compartment, prevent the induction of LTP and the movement of some of these molecules within spines and dendrites has been visualised during LTP [36,37]. The simplest locus for expression is therefore the same postsynaptic compartment. For presynaptic changes to occur there needs either to be an additional presynaptic induction signal, a diffusible retrograde messenger to convey the message back across the synapse or a signal conveyed by structural transsynaptic molecules, such as cadherins. No strong candidates have yet emerged.

Broadly speaking, evidence that LTP is expressed as a postsynaptic alteration comes in two categories. First, there is evidence that LTP is not presynaptic - and therefore by default is assumed to be postsynaptic. Evidence of this sort is inherently weakened by its reliance on assumptions that the experiment would have detected a presynaptic change had it occurred. Even the best controlled experiment cannot exclude all eventualities which could militate against a presynaptic change being observed, as is well illustrated by the first experiment considered in the next section. The second type of evidence, which we regard as much stronger, is where a postsynaptic change is directly observed. These types of evidence will be discussed in turn.

### Evidence that LTP is not presynaptic

In an early experiment that appeared to demonstrate that LTP was not expressed presynaptically, LTP was found to be accompanied by a specific increase in AMPA receptor-mediated EPSCs with no change in NMDA receptor-mediated EPSCs [38]. If LTP is expressed by an increase in synaptically evoked glutamate, it was argued, both components of the evoked response should be potentiated in parallel. Since this was not the case, LTP cannot be presynaptically mediated and expression must therefore be postsynaptic. However, this conclusion is potentially undermined by the fact that NMDA receptors have a markedly higher affinity for glutamate than AMPA receptors, and so are likely to be closer to saturation under basal conditions and thus less able to report changes in glutamate concentration. Moreover, this particular experiment also suffers from a lack of reproducibility. Numerous studies have reported that NMDA receptor-mediated EPSCs are able to sustain LTP (e.g., [39-42]). What can be concluded, however, is that under certain circumstances LTP is expressed in a manner that is not faithfully reflected by parallel increases in AMPA and NMDA receptor-mediated EPSCs.

A second type of experiment exploited the use-dependent nature of certain blockers as an indicator of p(r). The assumption here is that the drug is only able to gain access to its binding site when the channel is open, a condition which requires transmitter to bind to the receptor. Thus the rate of reduction of the EPSC reflects the distribution of p(r) in the axon terminals of the population of fibres being stimulated. Assuming presynaptic expression, the rate of decline of the EPSC in the presence of the activity-dependent blocker should be accelerated relative to pre-induction rates. No such result was observed in the initial experiment, using the use-dependent NMDA receptor blocker MK801 [43], but again there has been a lack of reproducibility, with evidence for an acceleration of the rate of decline after the induction of LTP [23]. The experiment has also been performed using GluA2 knockout mice, where the AMPA receptor-mediated EPSC is susceptible to a use-dependent block by a spermine derivative. Again, there was no evidence for an increase in p(r) following the induction of LTP [44], but this form of LTP involves a significant NMDA receptor-independent component [45].

The most compelling negative experiment has used glial cells to report changes in glutamate concentration. In these experiments, reproduced in three different laboratories [46-48], the electrogenic nature of glutamate uptake is exploited as a sensitive, real-time measure of its neuronal release. In no case was LTP associated with an increase in the transporter current. This experiment has positive controls: post-tetanic potentiation and paired-pulse facilitation, both of which are presynaptic phenomena, are mirrored by an increase in the electrogenic uptake current, and an increase in glutamate uptake is seen in mossy fiber LTP [48], which is generally agreed to be presynaptically expressed. On the face of it, the glial uptake experiments present a formidable case against a substantial presynaptic component to LTP at CA1 synapses. Can these findings be reconciled with a presynaptic mechanism? One possibility is that PPF generates a larger increase in glutamate release than occurs in LTP, leading to spillover and glial uptake, whereas in LTP any increased release is accommodated by neuronal transporters. (Note that ‘increased release’ in this context refers to a population of presynaptic terminals; at the level of the individual terminal what changes is the probability of release, and while there may be a greater than normal change in the concentration of released glutamate in the synaptic cleft during the rapid application of two stimuli in PPF, this will not in general be the case in LTP where single shocks are delivered at intervals of many seconds). Consistent with the possibility that an increase in glutamate release due to an increase in p(r) during LTP is accommodated by local neuronal uptake, neuronal glutamate transporters, but not glial transporters, exhibit increased activity during early LTP [49].

### Quantal Analysis

As discussed above, the attempts to obtain evidence for a postsynaptic expression of LTP via the application of quantal analysis using purely electrophysiological techniques have been difficult to interpret [50]. However, some observations are more simply explained by postsynaptic alterations. For example, an analysis of spontaneous and miniature EPSCs revealed an increase in amplitude but not frequency of these events [51]. A similar conclusion was reached when Sr^2+^ was used in place of Ca^2+^ in the medium so that individual quanta could be resolved following synaptic stimulation [52]. In addition, early in development (P6 - P12) a form of LTP was observed in some, but not all, neurons where changes were seen in potency but not success rate [12].

### Silent synapses

Postsynaptically silent synapses are defined as synapses that lack AMPA receptors but express NMDA receptors and so are silent at negative resting potentials. In fact, at rest (−60 to −70 mV) there is a finite NMDA receptor conductance and so such synapses will exhibit a low level of NMDA receptor activation (and hence are not strictly silent). Silent synapses can be unsilenced following the induction of LTP by the presumed insertion of AMPA receptors, leading to AMPA receptor-mediated EPSCs [53,54].

Studies by Emptage and colleagues established an interesting state-dependence in the expression mechanism of LTP in organotypic cultures. Postsynaptically silent synapses responded to LTP-inducing stimulation by insertion of synaptic AMPA receptors, without change in p(r); a subsequent tetanus to the now non-silent synapse produced further potentiation via an increase in p(r) [55]. The implication of this result is that LTP at immature, silent synapses is postsynaptically mediated, but that subsequent potentiation is expressed presynaptically.

It is probable that the unsilencing of silent synapses makes its major contribution to LTP early in life, since they are particularly prevalent early in development, declining rapidly during the first weeks of life [56].

### Sensitivity Experiments

One way to circumvent the uncertainties regarding the quantal nature of glutamate release at central synapses is to probe the postsynaptic sensitivity of potentiated synapses using the exogenous application of an agonist. This makes the reasonable assumption that any postsynaptic change would be detectable as an increase in the postsynaptic response to an exogenous agonist that acted mainly, or exclusively, on receptors at potentiated synapses. When this experiment was first attempted, no increase in sensitivity to glutamate was observed [57]. However, glutamate is protected from reaching synaptic receptors by powerful uptake mechanisms, which themselves generate an electrogenic signal in both glial cells and neurons and which will tend to mask the receptor-gated response. So this negative result may have a technical explanation. When the experiment was repeated using exogenous ligands for the AMPA receptor, increases in sensitivity were consistently observed [58]. The initial experiments used ionophoresis to apply the agonist. In some of these experiments the diffusion of agonist was limited to a few microns but may still have acted upon extrasynaptic as well as synaptic AMPA receptors. In subsequent experiments caged glutamate was uncaged by laser illumination in the vicinity of individual spines. An increase in sensitivity, as well as an increase in the size of the spine was reported, clearly a postsynaptic change [59]. Here, LTP was induced by repetitive pulses of light to uncage glutamate, and so it cannot be assumed that the same result would be obtained by repetitive stimulation that also involved the presynaptic side of the synapse. One difference in outcome of these different types of sensitivity experiments was the time-course of the development of the increase in sensitivity. Uncaging produced a more immediate potentiation than ionophoresis for reasons that are currently unclear.

There are a variety of ways in which a postsynaptic component of LTP could be expressed. Changes down-stream of AMPA receptors, such as alterations in intrinsic conductances leading to more efficient charge transfer to the soma, is one such mechanism that has experimental support [60]. More directly, AMPA receptors themselves could express LTP through an increase in their number at the postsynaptic density, or through a change in their biophysical properties.

### Changes in AMPA receptor properties

Broadly speaking intrinsic changes in receptor channel properties could reflect changes in (i) p(o) (the probability of channel opening on binding glutamate), (ii) mean open-time and (iii) unitary conductance (γ). The combination of dendritic recording, to improve the fidelity of the voltage-clamp, and non-stationary fluctuation analysis enabled these parameters to be estimated before and after the induction of LTP [61]. No evidence for a change in mean open-time was observed in these experiments but an increase in γ was detected that was sufficient in the majority of neurons sampled in 2-week-old rats to account entirely for the LTP observed. AMPA receptors have multiple conductance states [62,63] and the value of γ that is estimated by non-stationary fluctuation analysis is the weighted mean of these different conductance states.

The time that AMPA receptors occupy the various conductance states can be regulated both by phosphorylation of the receptor [64] and by the concentration of glutamate that they sense [65]. Consequently, the increase in γ observed in the LTP experiments could have both pre- and post-synaptic explanations. Regarding the former, any mechanism that results in more glutamate reaching the receptor could increase γ; for example, an increase in the amount of glutamate released from a vesicle or a reduction in local glutamate uptake. Phosphorylation of the receptor is the simplest postsynaptic explanation for an increase in γ. In this regard, CaMKII is activated during LTP, phosphorylates GluA1 on ser831 and this modification can lead to an increase in the time that AMPA receptors occupy the higher conductance states [66]. The ability of CaMKII to regulate AMPA receptor γ depends on the subunit composition of the receptor and the associated auxiliary subunits [67]. Since γ of both homomeric GluA1 and GluA1/GluA2, complexed with certain TARPs, can be regulated by CaMKII this is a plausible explanation for those cases where LTP is associated with an increase in γ. Yet another explanation for the increase in γ is the lateral movement of AMPA receptors from a region that senses a lower concentration to one that senses a higher concentration of glutamate. This would constitute one of several mechanisms whereby the trafficking of AMPA receptors is modified, a topic into which we now delve deeper.

### Alterations in AMPA receptor trafficking

Several different lines of evidence have supported the idea that LTP involves an increase in the number of AMPA receptors at synapses.

One approach has been to use antibodies that recognize AMPA receptors on the surface of living neurons. The first antibody to be developed for this purpose recognizes an epitope on the N-terminus of GluA1. In cultured hippocampal neurons this antibody was initially used to compare the cell surface and total distributions of GluA1-containing AMPA receptors and provided anatomical support for the existence of postsynaptically silent synapses [68]. In a form of chemical LTP, induced by treatment with glycine, there was an increase in the surface distribution of GluA1-containing AMPA receptors [69], consistent with the unsilencing of silent synapses or an exchange of GluA1-containing for GluA1-lacking AMPA receptors on the membrane surface. In a separate study, an antibody that recognizes all AMPA receptors, by binding to a common epitope, was used; in this study LTP was induced by very brief depolarization with K^+^ and resulted in the labeling of new sites, consistent with the unsilencing of silent synapses [70]. In both cases the increase in surface AMPA receptors depended on the activation of NMDA receptors. A refinement of this approach has been to use quantum dots, attached to these antibodies, to track the movement of single AMPA receptor subunits. Evidence has been presented that activation of NMDA receptors can result in the tethering of highly mobile AMPA receptor subunits to activated spines [71].

A second approach has been to use recombinant GFP-tagged AMPA receptors that have been modified to confer distinct electrophysiological properties following their insertion into the postsynaptic density. Using this approach it has been shown that LTP-inducing stimuli can drive recombinant AMPA receptors into synapses via a mechanism that involves NMDA receptors and CaMKII [72,73]. The tagging of AMPA receptors with a pH-sensitive variant of GFP [74], has also been used to demonstrate the insertion of AMPA receptors into the plasma membrane in response to a chemical LTP-inducing stimulus [75] and by glutamate uncaging [76].

A third approach has employed non-stationary fluctuation analysis to estimate the unitary conductance, γ, of AMPA receptors before and after LTP. In experiments performed on 2-week-old rats, an increase in γ was only observed in about two thirds of the neurons. In the remainder, there were increases in potency that is most simply explained by increases in the number of AMPA receptors expressed at synapses [61]. Surprisingly, during the first week of life there was a population of neurons in which LTP was associated with large changes in potency but paradoxically a reduction in γ [12]. This result is very hard to explain by anything other than a postsynaptic mechanism. It implies the synaptic incorporation of AMPA receptors with a lower γ than those that dominated under baseline conditions. This could occur, for example, if this form of LTP was associated with the insertion of GluA2-containing AMPA receptors into synapses that initially lacked these receptors. Consistent with this idea, hippocampal synapses acquire GluA2- lacking receptors before they acquire GluA2-containing ones [77].

A fourth approach has been to exploit the different properties of AMPA receptors with different subunit combinations. This was first used in the cerebellum to show the rapid, activity-dependent insertion of AMPA receptors that are relatively impermeable to Ca^2+^ into synapses that contain Ca^2+^-permeable AMPA receptors [78]. At hippocampal synapses, this situation is reversed; LTP involves the rapid insertion of Ca^2+^-permeable AMPA receptors at synapses that lack these receptors [79]. Ca^2+^-permeable AMPA receptors are any combination of subunits that lacks an edited GluA2 subunit; they confer upon the synapse a larger γ, inward rectification and sensitivity to block by polyamine derivatives, such as philanthotoxin. Any alteration of postsynaptic AMPA receptor subunits must, by definition, comprise a postsynaptic modification, though not necessarily an increase in number of receptors if the γ of the new receptors is higher. Supporting evidence for the selective insertion of GluA2-lacking AMPA receptors has been provided for LTP in cultured neurons, following a chemical LTP protocol [80]. However, LTP can occur without detectable alterations in the subunit composition of AMPA receptors [81] and the extent to which such changes contribute to the expression of LTP is presently unclear.

**Figure 4:**
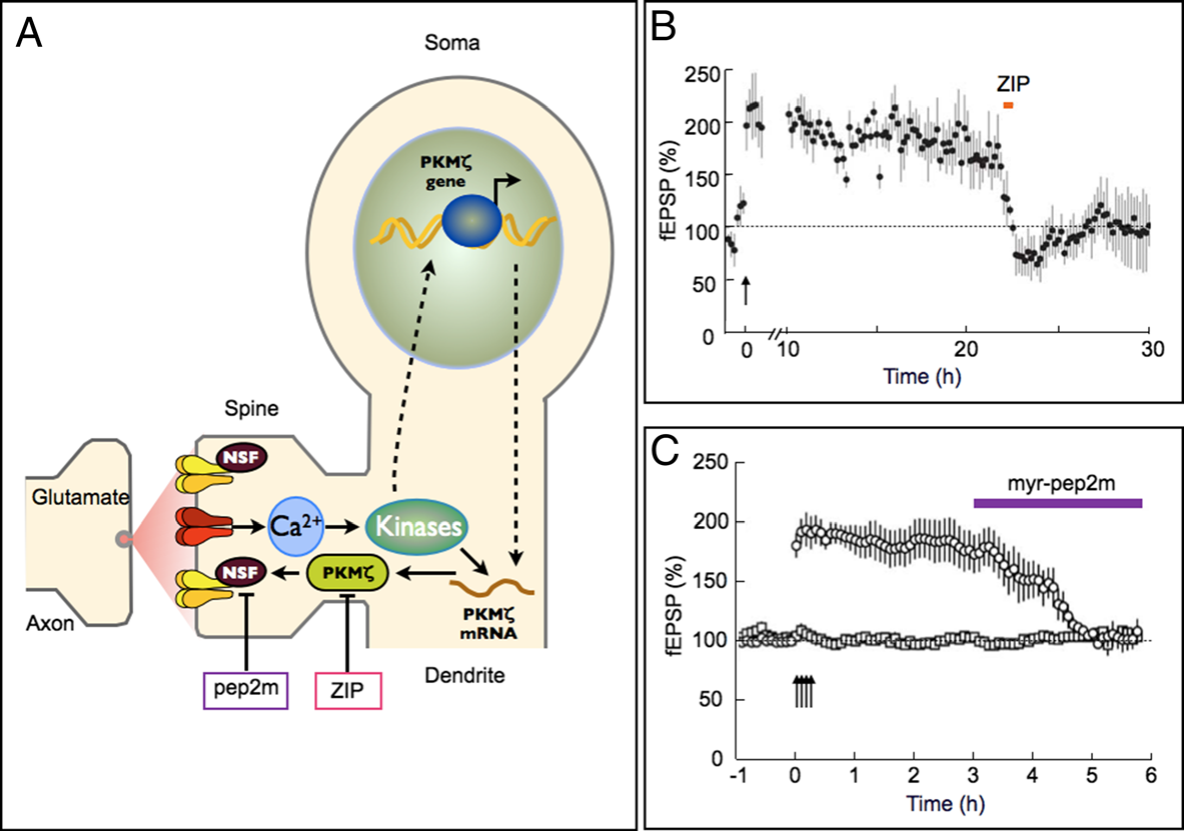
**A mechanism for postsynaptic AMPA receptor trafficking in LTP. A. **A scheme showing that NMDA receptor activation leads to the activation of PKMζ which stabilizes AMPA receptors at the synapse by promoting the interaction between the GluA2 subunit and N-ethylmaleimide-sensitive factor (NSF). **B. **ZIP, a peptide that inhibits the activity of PKMζ, inhibits the expression of LTP many hours after its induction. From [85]. **C. **Pep2m, a peptide that blocks the GluA2-NSF interaction, also inhibits the expression of LTP. From [83].

There has been considerable progress in understanding the mechanisms by which AMPA receptors are inserted, stabilized and removed from synapses. Some of this information has shed further light on the mechanism of expression of LTP. Most pertinently, it was found that an interaction between NSF and the GluA2 subunit stabilizes AMPA receptors at synapses [82]. A peptide, named pep2m, was designed that inhibited this interaction and was found to inhibit basal synaptic transmission, in an activity-dependent manner. The kinase PKMζ also stabilizes AMPA receptors at synapses; this seems to be due to a stabilization of the GluA2/NSF interaction [83] and a consequent suppression of clathrin-mediated endocytosis of AMPA receptors [84]. The relevance of this to the expression of LTP is the remarkable observation that blocking either the GluA2/NSF interaction, with pep2m [83], or inhibiting PKMζ activity [85] completely reverses LTP (Figure 4). This effect occurs over a large time-window (from tens of minutes to days and weeks) suggesting that the insertion and stabilisation of AMPA receptors is a particularly important expression mechanism of LTP in the adult animal. However, three recent publications have questioned aspects of this model. LTP can still be obtained when AMPA receptors are engineered to lack their C-terminal tails [86], and both LTP and learning are readily induced in PKMζ knockout mice [87,88]. While these studies may require a reconsideration of postsynaptic mechanisms of expression, they do not force a presynaptic conclusion. In both sets of studies, evidence for the importance of a postsynaptic contribution to LTP was obtained: LTP was impaired when the overall concentration of AMPA receptors was reduced below a critical level [86], and the peptide inhibitor ZIP still abolished LTP in PKMζ knockout mice [87,88], confirming that a ZIP-sensitive mechanism is required for the maintenance of LTP. It is possible, for instance, that a closely related kinase, PKMι/λ, has compensated for the loss of PKMζ. In our view, the mechanism outlined in Figure 4 remains the strongest molecular candidate to link synaptic activation of NMDA receptors to the alterations in AMPA receptor function that are associated with LTP.

In conclusion, multiple lines of evidence support the idea that LTP involves postsynaptic modifications, and shed some light on possible underlying mechanisms.

## Structural Alterations

The various pre and postsynaptic mechanisms described above could exist without the need for any structural alterations of synapses. However, there is good anatomical evidence that LTP is associated with changes in synapse morphology [89] and that these changes can occur rapidly following repetitive uncaging of glutamate at single dendritic spines [90]. But although growth and retraction of spines have been reported following LTP-inducing protocols [91] there have as yet been no reports of morphological changes following the induction of LTP at individually imaged spines.

## Discussion

There is no simple way in which the contradictory results on the expression mechanisms of early NMDA receptor-dependent LTP can be reconciled. Across the various studies we have discussed, there are multiple, potentially confounding factors. These include, but are by no means restricted to, differences in (i) the preparation (dissociated neurons, acute slice, organotypic slice, anaesthetised animal, freely moving animal), (ii) LTP induction protocols (chemical, tetanus, theta, pairing, spike-timing etc.) (iii) time after induction, (iv) interactions with other activity-dependent forms of synaptic plasticity, such as short-term potentiation (STP), that may occur in parallel with LTP, (v) developmental status of the animal, (vi) species and strain of the animal, (vii) animal housing (food, light–dark cycle, prior experience, level of stress), (viii) sex and hormonal status. The situation becomes even more complex when the diversity of expression mechanisms between different classes of synapses is also taken into account.

STP is of particular relevance to any discussion of the variables that influence the induction and expression of synaptic plasticity. The duration of STP (also sometimes termed transient LTP; t-LTP) is consistent under a given set of conditions but can vary considerably across studies (typically lasting between a few minutes and an hour) and, in some cases, may be absent altogether. STP has the remarkable property that it decays in an activity-dependent manner, so that in the absence of any stimulation to probe synaptic efficacy it can persist, in latent form, for many hours [92]. The presence and duration of STP can be predicted on the basis of the frequency of the stimuli used to induce and monitor synaptic plasticity; a high frequency tetanus followed by a low frequency of test stimulation favours STP. The relevance of this to the discussion of LTP expression mechanisms is that available evidence suggests that STP is primarily mediated presynaptically [92]. An STP-like phenomenon may account, therefore, for some of the reported studies that have provided evidence for presynaptic expression of LTP. A recent study has revealed differences in the NMDA receptor subtypes involved in the induction of STP and LTP [93]; it is possible therefore that two different forms of NMDA receptor-dependent with different induction and expression loci coexist at potentiation synapses.

**Figure 5:**
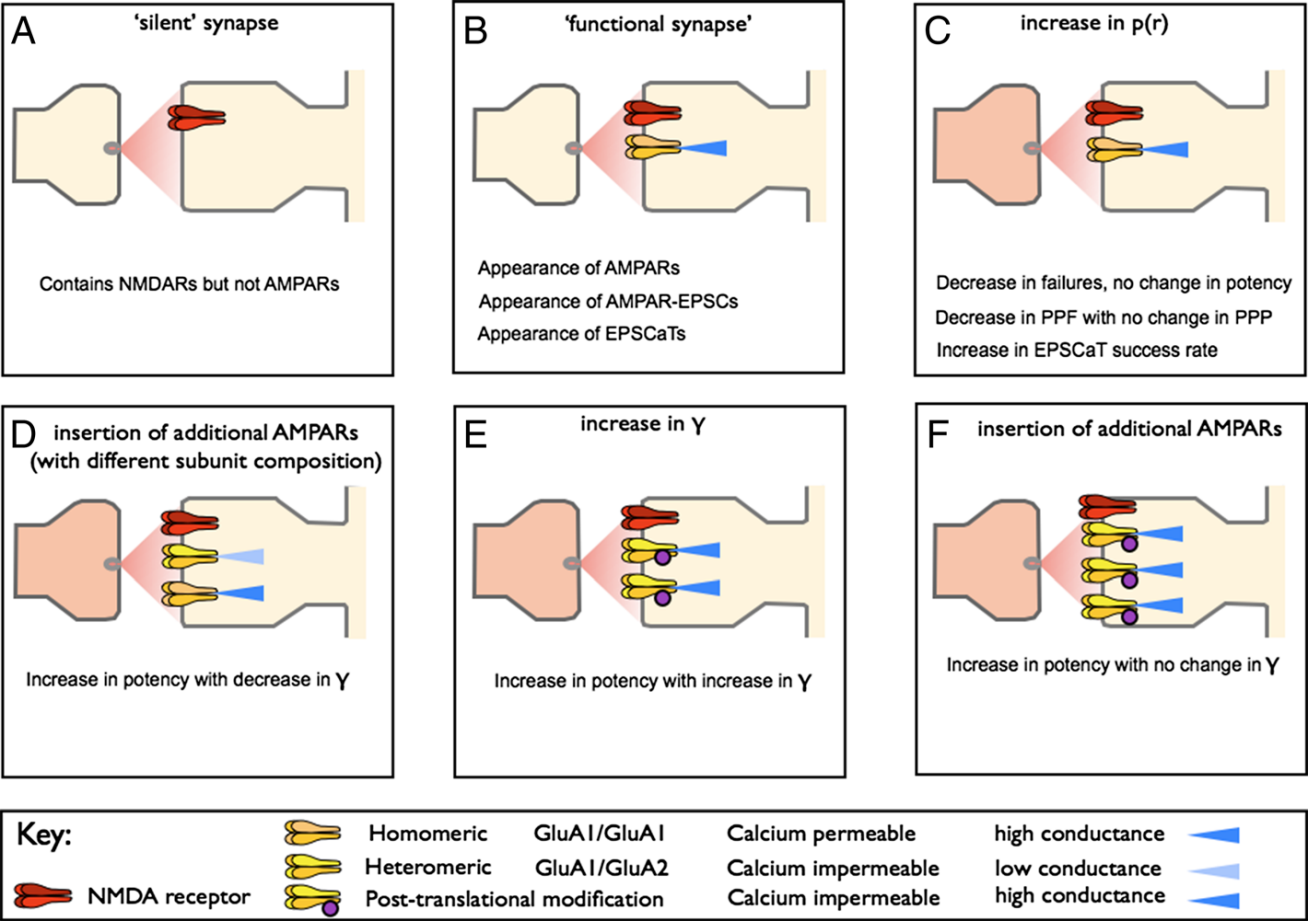
**Multiple mechanisms of expression of LTP. A. **Early in development synapses may acquire NMDA receptors before AMPA receptors (so-called silent synapses). **B.** The unsilencing of silent synapses is likely to involve the synaptic insertion of AMPA receptors. **C. **Complementary evidence exists showing that functional synapses can increase their synaptic strength via an increase in p(r) PPP = paired-pulse potency. **D. **There is also evidence for an increase in the quantal size due to the synaptic insertion of AMPA receptors exhibiting, paradoxically, lower γ. **E**, **F.** Increases in quantal size, due to an increase in γ (**E**) or in the number of AMPA receptors (**F**) have also also been observed.

Although any of the above factors alone or in combination may have an important influence on the outcome of the experiment, factors such as these cannot account for the entire heterogeneity of LTP expression mechanisms. This is demonstrated by studies in which more than one mechanism was observed in the same set of experiments. For example, in slices from young animals, expression mechanisms changed markedly between the first and second weeks of life, and even at the same developmental stage there was more than one LTP phenotype [12]; and in organotypic cultures, single visualised synapses were first unsilenced by a postsynaptic mechanism and then further potentiated by a presynaptic mechanism [29,55]. It is likely therefore that synapses in the hippocampus have the capacity to display multiple forms of NMDA receptor-dependent LTP (Figure 5), with the prevailing form at a given time depending on many, still to be identified, factors.

On the face of it the most convincing of the approaches discussed above is the imaging study of Enoki et al. [30], because this experiment yielded measurements of p(r) and quantal size at individual potentiated synapses. The results appear to deny a role to the postsynaptic side (no change in synaptic potency), while demonstrating a role for the presynaptic side (an increase in p(r)). Note, however, that these results, and indeed the great majority of experiments on expression mechanisms, only provide a snap shot of LTP at an early time point after induction. In the intact animal different mechanisms may support the expression of later stages of LTP, and here there is evidence from hippocampus [85] and amygdala [94] for postsynaptic changes.

It is also worth noting that most LTP studies have focussed on what happens to AMPA receptor-mediated synaptic transmission, since these receptors are the predominant mediators of fast synaptic transmission in the hippocampus, and elsewhere in the brain. However, in the intact animal high frequency discharges are a common firing mode and NMDA receptors contribute substantially to the synaptic response under these conditions. The synaptic response mediated by NMDA receptors is also capable of exhibiting LTP [39-42]. A full description of the expression mechanisms of LTP will also need to consider the plasticity of this major synaptic component.

Where does this leave us? Our strong feeling is that the evidence is too persuasive on both sides to assume that further experiments will reveal one or the other mechanism to be the outright victor in the eyes of an unbiased observer. We conclude that two expression mechanisms are available for NMDA receptor-dependent LTP, one presynaptic, resulting in an increase in p(r) without an increase in potency, and the other postsynaptic, resulting in a change in potency without an increase in p(r), and reflecting changes in the number and/or conductance properties of AMPA receptors. Either or both mechanisms could be induced in a given experimental situation, leading to cases where early LTP will be seen as entirely presynaptic, entirely postsynaptic or a combination of the two.

The property of LTP that most commends it as a cellular mechanism for encoding information is its longevity. The studies we have discussed so far reveal little about the mechanism of enduring LTP in the intact animal. Ultimately, the search for expression mechanisms will need to be conducted in the context of the neural networks subserving memory and cognition, processes that can potentially operate over a lifetime. Here progress will depend on techniques to study the plasticity and structure of single synapses in the freely moving animal. If and when agreement on a long-term expression mechanism based on structural changes is reached, pre and postsynaptic mechanisms will converge, and a controversy that has already continued for nearly four decades may finally be put to rest.
